# Supplementary material for: A cVLP-Based Vaccine Displaying Full-Length PCSK9 Elicits a Higher Reduction in Plasma PCSK9 Than Similar Peptide-Based cVLP Vaccines
Source: Vaccines (Basel). 2022 Dec 20;11(1):2. doi: 10.3390/vaccines11010002 (PMC9864010; doi:10.3390/vaccines11010002)
Supplement: Supplementary file 1 [file vaccines-11-00002-s001.zip › vaccines-2052348-supplementary.pdf]

Supplementary material

Table S1. Primers used for cloning.

**Primers for SpyC-PCSK9(156-227)**<br>**(Fusion of SpyC and PCSK9 by overlap-extension PCR).**<br>Template PCR1: SpyCatcher (Geneart)<br>Template PCR2: FL-PCSK9(35-692)-SpyT (Geneart)	
PCR1 FW_SpyC-PCSK9(156-227)	TTT CCA TGG GCC ATC ACC ATC ACC ACC ACG GTG CAA TGG TTG ATA CCC TG
PCR1 RV_SpyC-PCSK9(156-227)	CCA GGG GAT GGA GCT ACC ACC AAT ATG TGC ATC AC
PCR2 FW_SpyC-PCSK9(156-227)	GGT GGT AGC TCC ATC CCC TGG AAC CTC GAG
PCR2 RV_SpyC-PCSK9(156-227)	AAA GCG GCC GCT TAG TCG CAC TTG GAA GCC TGC CTG
**Primers for SpyC-PCSK9(210-226)**<br>Template: SpyCatcher (PCSK9 peptide was introduced by RV primer).	
PCR1 FW_SpyC-PCSK9(210-226)	TTT CCA TGG GCC ATC ACC ATC ACC ACC ACG GTG CAA TGG TTG ATA CCC TG
PCR1 RV_SpyC-PCSK9(210-226)	AAA GCG GCC GCT TAG CAC TTG GAA GCC TGC CTG TGG AAG CGG GTG CCA TCT TCC TCG GGC ACG GAG CTA CCA CCA ATA TGT GCA TC
**Primers for FL-PCSK9(35-692)R221S-SpyT**<br>**(introduction of R221S mutation by overlap-extension PCR).**<br>Template: FL-PCSK9(35-692)-SpyT (Geneart)	
PCR1 FW_FL PCSK9-SpyT	TTT GAA TTC GCC ACC ATG AAG CTG TGC ATC CTG CTG GCC GTG GTG GCC TTC GTG GGA CTG AGT CTG GGA CAG GAC GAG GAC GGC GAC
PCR1 RV_FL PCSK9-SpyT	GCC TGG GAG TGG AAG CGG GTG CCA TCT TCC TCG
PCR2 FW_FL PCSK9-SpyT	CTT CCA CTC CCA GGC TTC CAA GTG CGA CTC CCA C
PCR2 RV_FL PCSK9-SpyT	TTA AAG CGG CCG CTT ATT TAG TGG GC

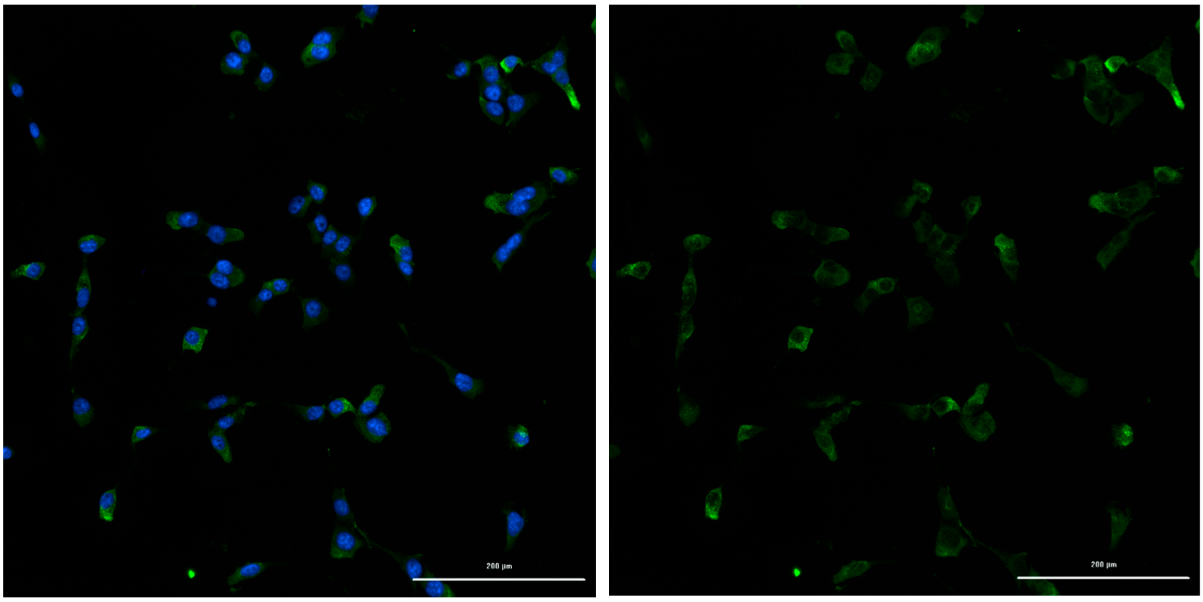

Supplementary Figure S1 LDLR staining on Hepa1-6 cells. Hepa1-6 cells incubated with media (control) for 4h at 37°C. Cells were stained with DAPI (blue) and anti-LDLR-FITC (green). Representative cells are shown as merged pictures (left) or FITC signal alone (right). The size bar is 200µm

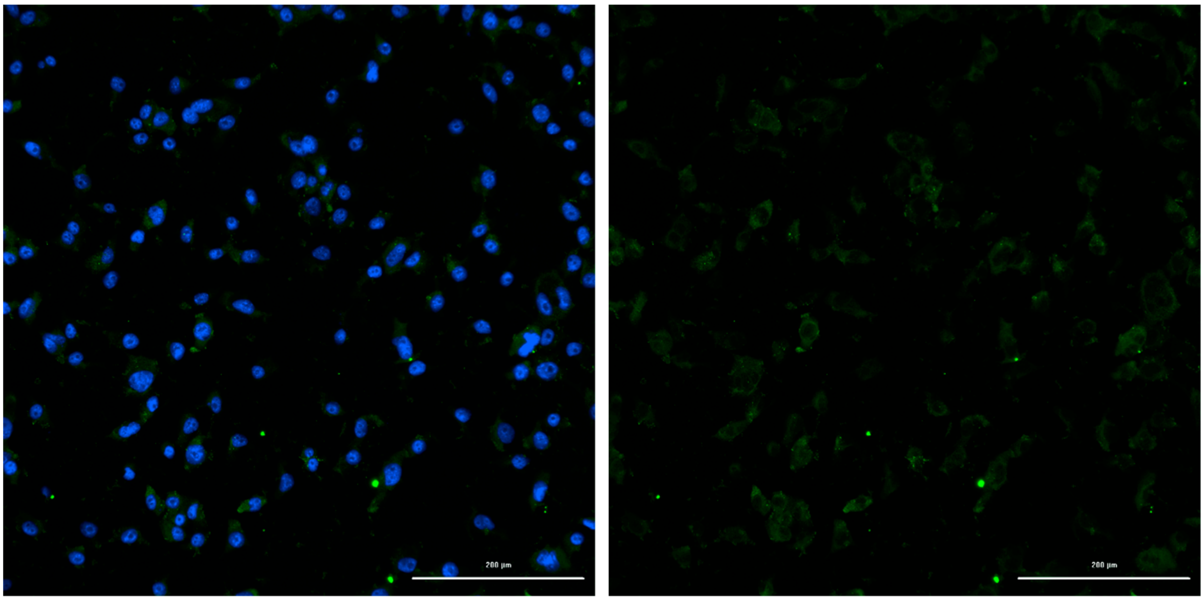

Supplementary Figure S2 Biological activity of PCSK9 displayed on cVLPs by measuring LDLR staining on Hepa1-6 cells. Hepa1-6 cells incubated with 100nM PCSK9-SpyT displayed on cVLPs for 4h at 37°C. Cells were stained with DAPI (blue) and anti-LDLR-FITC (green). Representative cells are shown as merged pictures (left) or FITC signal alone (right). The size bar is 200µm

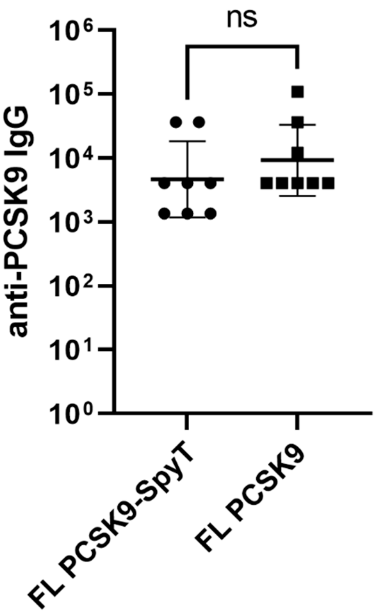

Supplementary Figure S3 Comparison of ELISA coat. Serum from mice vaccinated with cVLP-PCSK9 FL (n=8) was tested against FL PCSK9-SpyT (produced in S2 cells) or recombinant FL PCSK9 (produced in HEK293 cells) (Abcam). The end-point titer (determined using a cut-off of OD450>0.2) was plotted. Statistical analysis was performed using a non-parametric, two-tailed Mann-Whitney T test (adjusted p value < 0.05 was accepted as significant).
